# Long-term outcomes of a father-daughter program for sport participation, activity, wellbeing, and gender equity: a 3-8-year mixed-methods follow-up

**DOI:** 10.1186/s12889-026-26287-7

**Published:** 2026-01-30

**Authors:** Philip J. Morgan, Jaqueline A Grounds, Rosslyn O’Connor, Daniel R Lee, Lee M Ashton

**Affiliations:** 1 Global Sport and Movement Collaborative, College of Human and Social Futures, School of Education, University of Newcastle, University Drive, Callaghan, NSW Australia; 2 Active Living and Learning Research Program, Hunter Medical Research Institute (HMRI), Lot 1 Kookaburra Circuit, New Lambton Heights, NSW 2305 Australia; 3 Department of Population Health, Hunter New England Local Health District, Newcastle, NSW Australia

**Keywords:** Fathers, Girls, Physical activity, Parenting, Long-term follow-up

## Abstract

**Background:**

Dads and Daughters Exercising and Empowered (DADEE) is a program that engages fathers/father-figures to improve their primary-school aged daughter’s physical activity levels and social-emotional wellbeing. The holistic benefits of this program have been previously reported at post-program, 9-months and 12-months post-baseline. This study aims to investigate the long-term impact of the program on the fathers, daughters and broader family unit when followed-up between 3–8-years after program completion.

**Methods:**

A mixed-methods design was employed, including online survey data and one-on-one semi-structured interviews with fathers that attended the program between 2015 and 2020. Both survey and interview questions focused on fathers’ perceptions of any long-term impact of the program on themselves, their daughter, and their family. The survey also explored daughters’ current participation in sport and physical activity. All information about impacts on daughters and the wider family unit was obtained through father proxy report. Descriptive analyses were conducted for quantitative data, while the qualitative dataset was analysed independently using an inductive thematic analysis.

**Results:**

A total of 197 fathers (50% of those invited) completed the online survey (fathers; 47.5 ± 4.9 years, daughters; 14.4 ± 2.4 years), while a random selection of 24 fathers completed interviews (fathers; 46.9 ± 4.9 years, daughters: 14.2 ± 2.9 years). For daughters, three key themes emerged as prominent sustained impacts: *D1. More sports participation and active lifestyle*, *D2. Enhanced social-emotional wellbeing* and *D3. Awareness and ability to overcome gender inequities*. For fathers, four key themes emerged: *F1. Gender equity advocates*,* F2. Prioritising the father role*,* F3. Empowering daughters to stay active* and *F4. More active lifestyle*. Two key themes emerged for the father-daughter dyad: *FD1. Increased co-physical activity* and *FD2. Closer relationship in the teenage years*, while two themes emerged for the wider family unit: *Fam1. Gender equity advocates* and *Fam2. Establishing new family routines.*

**Conclusions:**

This novel physical activity and wellbeing program targeting fathers and daughters has led to perceived long-term effects (between 3 and 8 years since program completion) for fathers, daughters and the family unit. The evidence-based strategies implemented in the program can inform design and delivery of more effective family-based lifestyle programs, with potential to achieve long-term, holistic benefits.

**Trial registration:**

Australian New Zealand Clinical Trials Registry: ACTRN12623000626662. Prospectively registered: 07/06/2023.

**Supplementary Information:**

The online version contains supplementary material available at 10.1186/s12889-026-26287-7.

## Background

Physical inactivity in girls is a major global issue [1]. Girls are significantly less active than boys at all ages due to a range of socio-cultural factors [1–4]. A lack of sports skills amongst girls, with less than 5% of primary school aged girls having mastered fundamental sports skills [5], contributes to high rates of sports drop out and compounds physical inactivity across the lifespan [1]. Boys have far higher rates of sports participation [6], and are less likely to drop out [7]. Fathers are major influencers on the physical activity and sports participation of their children [8]; however, boys receive more attention from their fathers in the physical activity and sporting domains [9, 10].

‘Dads and Daughters Exercising and Empowered’ (DADEE) (currently known as Daughters and Dads Active and Empowered) was the world’s first program to target fathers/father-figures (referred to as ‘fathers’ hereafter) as the agents of change to improve their daughters’ social-emotional wellbeing and physical activity levels [11]. By drawing on the unique benefits of the father-daughter relationship, the program innovatively addressed alarming inequities in sport and physical activity for girls and improved the physical activity levels of both [11, 12]. DADEE was designed to motivate fathers to be gender equity advocates and physical activity leaders for their primary school aged daughters so that they too may reap the lifelong health benefits derived from both the father-child relationship and engagement with physical activity and sports. The 9-week face-to-face program achieved this in a range of novel ways. Firstly, fathers attended a workshop where they were presented with evidence-based information about; their importance in their daughters’ lives, the challenges that girls’ face in the modern world, how to increase the physical activity, sport skills, social-emotional skills and wellbeing of their daughters, and how to optimise their parenting. Fathers then attended eight weekly sessions with their daughters (30 minutes education, 60 minutes practical). The program utilised reciprocal reinforcement with fathers and daughters acting as role models to help one another become more physically active. Education content each week covered a specific topic, such as physical activity, female role models, sport skills, and screen time, along with one of the following key social-emotional constructs; self-control, persistence, resilience, kindness, bravery, positivity, critical thinking, and self-reliance. A key aspect of the program was teaching the critical thinking skills necessary to identify, navigate and challenge gender prejudice that infiltrates all aspects of girls’ lives, particularly in the sporting and physical activity domains. Each week also included practical sessions divided into three sections: rough and tumble play, fundamental movement skill practice (kick, catch, strike, bounce, overhand throw, and underhand throw) and fun aerobic and muscular fitness activities. A more detailed outline of the program’s structure and elements is published elsewhere [11].

The program has been extensively researched and demonstrated numerous improvements in fathers’ and daughters’ physical and mental health. In a 2015 efficacy randomised controlled trial (RCT) with 278 participants, the program led to improvements at 9-month follow-up in daughters’ physical activity levels, fundamental movement skills, screentime habits [11], and social emotional well-being [13]. Fathers’ physical activity and parenting practices also improved [11] and the father-daughter relationship was enhanced [13]. In a 2016 effectiveness RCT with 344 participants, each of these improvements were again evident at 3-month follow-up, alongside improvements to daughters’ self-esteem [12, 14]. To test the sustained impact of the program at 12-month follow-up, a further 542 participants were involved in a non-randomised trial between 2017 and 2019. Each of the previously mentioned improvements were evident once more [15]. Qualitative research with fathers on their perceptions of the program’s impact has triangulated these quantitative findings. Key qualitative themes on the program’s impact after completion included improvements in daughters’ social-emotional well-being, the father–daughter relationship, co-parenting and father’s parenting skills. Fathers also expressed greater involvement with their daughters and enhanced family dynamics. Additionally, they improved their knowledge and understanding of gender stereotypes and gender bias [16].

While the 12-month findings were promising and demonstrated the program had sustained benefits for participants’ physical and mental health and family relationships, it is not known whether the program instilled benefits in the longer term and if the knowledge, skills and behaviour improvements were maintained. Long-term follow-up is widely recognised as important in health intervention research [17], particularly for interventions targeting the health behaviours of young people [18] and families [19]. It helps assess the sustainability of intervention effects, provides clearer estimates of benefits and costs, allows for intervention refinement based on outcomes, tracks behavioural, social, familial, and developmental changes, and identifies key periods for prevention [18, 20]. However, long-term follow up, particularly beyond 12 months, is severely lacking in interventions targeting: children’s physical activity in families [19, 21], wellbeing [22, 23], fathering [24], men’s health [25, 26], and girls’ physical activity [27, 28]. Therefore, the current study sought to explore the impacts of the DADEE program up to eight years after participation. Exploring these longer-term program impacts is of particular importance given the daughters followed up were now mostly teenagers and, for girls, the teenage years see increased sports drop out [29], sedentary behaviour [30], body image issues [31], psychological distress [32, 33] and difficulties in relationships with fathers [34]. The primary aim of this paper is to investigate the long-term impact of the DADEE program on the fathers, daughters and broader family unit when followed-up between 3 and 8 years post-program completion.

## Methods

### Study design

A mixed-methods design aligning with an explanatory sequential approach [35] was used to explore the long-lasting impacts of the program. Details of the program have been published elsewhere [11, 12]. Firstly, quantitative data were collected through an online survey of fathers who previously participated in the program between 2015 and 2020. Secondly, qualitative data were obtained through online interviews with a randomly selected subsample of fathers who completed the survey, to minimise bias and provide a deeper understanding of the long-lasting impacts of the program on themselves, their daughters and their wider family unit. The study received institutional approval from the Human Research Ethics Committee (H-2014–0330) and was prospectively registered with the Australian and New Zealand Clinical Trials Registry (ACTRN12623000626662).

### Participants

#### Target population

The study population comprised fathers and father-figures who completed the program between 2015 and 2020. Throughout this paper, ‘fathers’ refers to both biological fathers and other father-figures who completed the program (e.g. stepfathers, adoptive fathers).

#### Eligibility criteria

Fathers were eligible to participate if they attended at least 70% of the program sessions. Participants were excluded if they had not provided permission to be included in the alumni contact list or had opted out.

#### Sampling and recruitment

##### Quantitative (Survey)

Eligible fathers were contacted via email by a member of the research team, with an information statement seeking their interest and a link to provide consent to participate in the study. Once consent was provided, individuals were then directed to the online survey. Fathers who completed the survey were offered the chance to enter a modest voucher draw (with the chance to win one of three $100 vouchers) as an acknowledgement of their time. The incentive was non-coercive and unlikely to influence participation or consent, and Institutional ethical approval was provided for this common approach to survey completion. All included survey participants provided written informed consent.

##### Qualitative (Interviews)

All survey completers were invited to participate in a follow-up interview, via an expression of interest question within the survey. Interested fathers were allocated into a stratum based on (1) the current age of the eldest daughter who first participated in the program and (2) the year they first participated. Within each stratum, a computer-based random number generator was used to randomly select fathers to be contacted for interview scheduling. If a selected father did not respond after receiving the initial email invitation and two follow-up emails, the next randomly generated ID within the same stratum was contacted. Interviewees received a $50 a gift voucher to acknowledge their time. All interview participants provided written informed consent.

### Data collection

#### Online survey

Online survey responses were collected between 18th June 2023 and 5th October 2023 and completed by fathers. The online survey was managed using REDCap electronic data capture tools [36, 37]. Survey questions included both validated questions and questions developed for this study by members of the research team. The survey included questions relating to their daughters’ participation in sport and physical activity, and fathers’ perceptions of the long-term impacts of the program on themselves, their daughter and their family (Table 1). The survey took approximately 15 min for participants to complete.

**Table 1:** Overview of measures in the online survey, completed by fathers

Measure	Description
*Daughters’ MVPA (self-reported days meeting physical activity recommendations- days/week)*	• **Measurement tool**: A single item question from the Australian Bureau of Statistics *‘Australian Health Survey’* [38], previous research has shown this single-item measure to be a reliable and valid assessment of youth physical activity [39]. • **Metrics/questions**: Single item question: *“On how many of the past 7 days did your daughter engage in sport*,* physical activity or active play for a total of at least 60 minutes? Some examples include playing soccer*,* netball*,* basketball*,* rugby league or union*,* Australian Rules football*,* swimming*,* walking or riding a bicycle to or from school*,* skipping*,* running*,* rollerblading*,* dancing or any activity that made your daughter huff and puff.”*
*Father-daughter co-physical activity (days/week)*	• **Measurement tool**: 2-items adapted from the Youth Media Campaign Longitudinal Survey [40]. • **Metrics/questions**: Fathers reported on days per week they were physically active with their daughter on-on-one and with their daughter and one or more other family members.
*Daughters’ sports/physical activity participation in the last 12-months.*	• **Measurement tool**: Multiple item question designed for this study. • **Metrics/questions**: *“In the last 12 months*,* has [daughter] participated in any of the following organised sports and physical activities?* Fathers could select all sports/activities that apply via checkbox (multiple answers). Response options included 19 common sports from AusPlay data [41], with open response field for anyone selecting ‘other’.
*Daughters’ sport/physical activity participation level*	• **Measurement tool**: Multiple item question designed for this study. • **Metrics/questions: “***Based on your previous answers*,* please indicate the highest level at which [daughter] currently participates in the following sports or physical activities”* Fathers could select any one of the following for each sport the daughter participates in: 1. Home/local/park/social with friends and family 2. School based 3. Community club member 4. Regional or state representative competition. 5. National or international representative competition.
*Daughters’ continued participation*,* drop out and new sports/activities since program participation.*	• **Measurement tool**: Multiple item question designed for this study. • **Metrics/questions**: *Since participating in the Daughters and Dads program in [program_year]*,* please indicate if [daughter] has continued participation*, taken up any new sports/activities or dropped-out* of any of the following sports/activities” Response options included 19 common sports/activities from AusPlay data [41], with open response field for anyone selecting ‘other’. *For those selecting dropped out, an additional question was asked relating to reasons for dropping out.
*Impact of COVID-19 pandemic on daughters’ sport/activity participation*	• **Measurement tool**: Single item question designed for this study. • **Metrics/questions: “***Has the COVID-19 pandemic had any impact on [daughter’s] participation in organised sport and/or physical activity?”* Fathers could select ‘yes’, ‘no’ or unsure.
*Long-term impact of the program*	• **Measurement tool**: Multiple item question designed for this study. • **Metrics/questions**: Fathers were asked if the program has had a long-lasting positive impact on key program outcomes for themselves and their daughter (e.g., physical activity, parenting, father-daughter relationship, mental health, awareness of gender inequity). Responses were on a 5-point Likert scale where strongly disagree = 1 and strongly agree = 5.

If a father participated in more than one program and/or with more than one daughter concurrently he was asked to answer with reference to the first program he participated in and the eldest daughter he participated with during that program to reduce participant burden and confusion (this also applied to the online interviews). Demographic data for the fathers were also collected, including year and month of birth, highest level of education, marital status, employment status, year and month of daughter’s birth, relationship to daughter, daughter’s current school year or tertiary educational level if applicable.

#### Semi-structured interviews

One-on-one semi-structured interviews were conducted between 17th August 2023 and 26th October 2023 and lasted on average 26 min (range: 12 min to 44 min). Interviews were conducted online, using Zoom Software (Zoom Video Communications Inc. San Jose, California, USA). Audio from the interviews was recorded and transcribed verbatim. Interviews were conducted by three members of the research team (2 female research assistants and 1 male postdoctoral project officer), who were all parents. No relationship between the interviewers and participants was established prior to the study and only the interviewer and participant were present during interviews. Prior to interview commencement, participants were sent an information statement explaining the interview topic and this was reiterated by the interviewer at the start of each interview. A total of 12 questions were developed by members of the research team, but only the responses to 10 questions are included in this paper. To note: the opening/ice breaker question and a question relating to immediate impact (rather than long-term impact) were not included in the analysis for this paper. Interview questions aimed to explore any long-lasting impacts of the program on fathers, daughters, or the wider family unit. Probing techniques to facilitate elaboration on responses were also used. At the end of the interview, participants were asked to provide any additional comments that may have been missed. The Interview guide can be found in Additional File 1.

### Data analysis

#### Online survey

Descriptive analyses (i.e., percentage and frequency counts) were conducted to assess the long-term impact of the program on key outcomes (outlined in Table 1). Inferential analyses were not possible, as follow-up measures differed from those used 3–8 years earlier, which were validated for primary-school-aged children, whereas most daughters were now teenagers or young adults. Health and physical activity determinants change substantially during adolescence [42, 43], making direct comparisons over time difficult. Therefore, quantitative findings were used primarily to support and triangulate qualitative results, rather than to determine causal effects or magnitude of impact. All analyses were conducted using STATA v17.0 [44].

#### Qualitative interviews

The transcribed dataset was analysed using an inductive thematic analysis [45] by an experienced, independent qualitative researcher with over 20 years’ experience in qualitative research, who had not been involved in any aspect of the study, including design or data collection. This approach to analysis was chosen due to the importance placed on allowing participants’ experiences to shape the thematic structure. QSR NVivo Version 12 [46] was used to assist with the organisational aspects of the analysis.

The first phase of analysis consisted of a thorough review of the entire dataset, in which notes were made of the types of long-term impacts the participants talked about for themselves, their daughters and their families since program participation. This data immersion was used as a foundation for the second stage of analysis, in which in vivo codes were generated from across the entire dataset. This formed the basis for a preliminary thematic structure which was discussed with the wider research team who had familiarity with the dataset, to ensure that the coding and thematic structure reflected the participant experiences as interpreted by research staff with different levels of project involvement. This process of investigator triangulation enhanced the credibility of findings by incorporating multiple perspectives on data interpretation and reducing individual researcher bias.

On the basis of this consultative phase, the dataset was coded according to the final coding structure and reviewed for consistency both within each code and across the entire dataset. Themes and sub-themes were generated, with all data allocated to each theme and sub-theme once more reviewed for consistency, to ensure that each theme represented a unique contribution to the story of the overall dataset. Thematic summaries were formulated with extensive use of representative verbatim quotes from across the dataset which provided thick description and an important link between the reported findings and lived experience, enabling readers to assess the transferability of findings to other contexts.

## Results

### Flow of participants

**Fig. 1:**
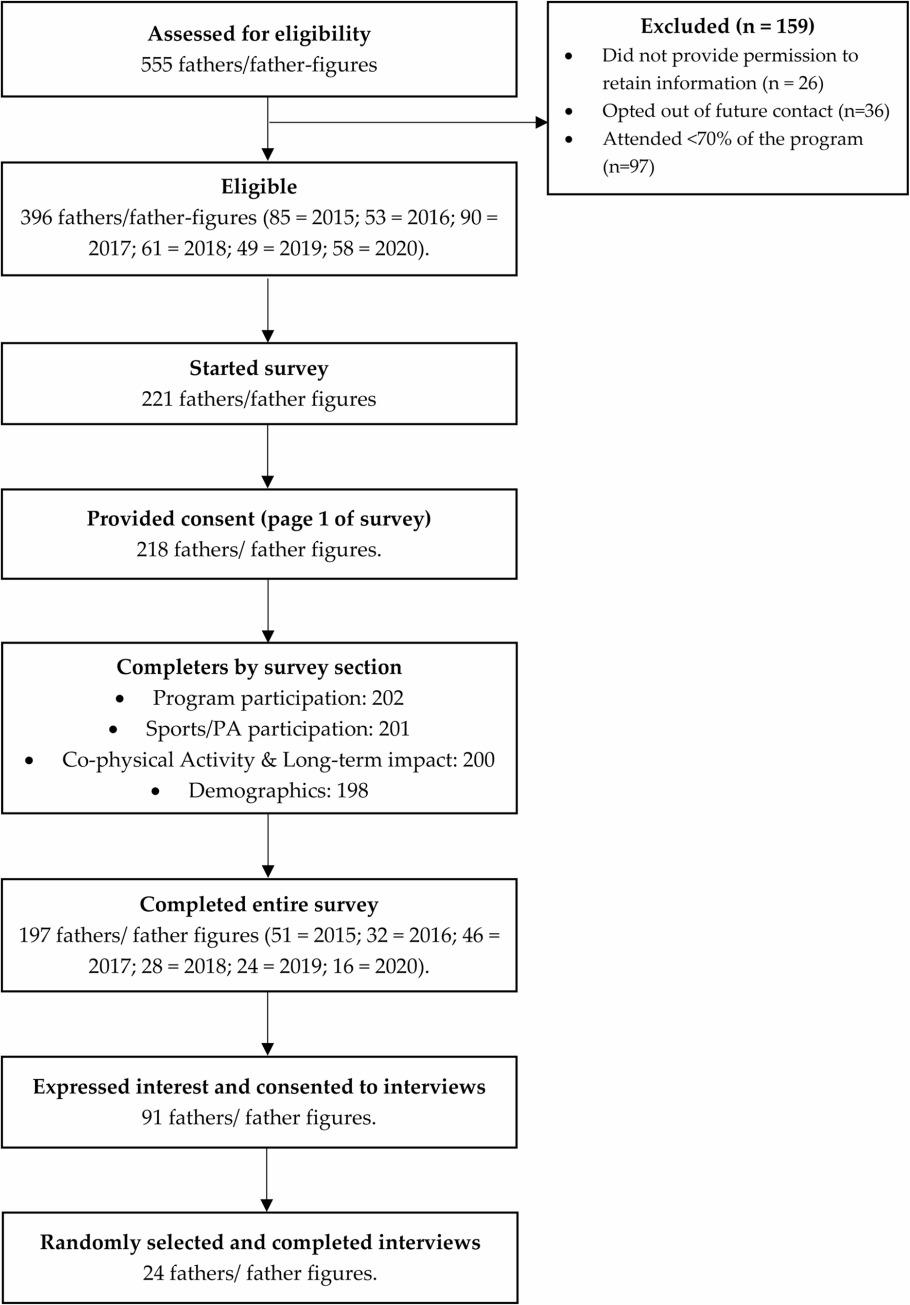
Flow of participants

A total of 396 fathers (85 from 2015; 53 from 2016; 90 from 2017; 61 from 2018; 49 from 2019; and 58 from 2020) were eligible for this study and were invited to participate. Of these, 221 (56%) fathers started the survey, 218 (55%) provided consent (page 1 of survey) and 197 (50%) completed all questions. Of the 197 survey completers, 46% (*n* = 91) were open to participating in an interview and provided consent to do so. A total of 24 fathers were randomly selected from these and completed interviews (Fig. 1).

### Program participation

For fathers undertaking the survey (*n* = 202), there was a greater response rate from those who undertook programs in the earlier years (percentage of participants from those eligible; 60% in 2015, 60% in 2016, 56% in 2017, 48% in 2018, 49% in 2019 and 28% in 2020). Most fathers enrolled one daughter (*n* = 173, 85.6%) when first participating in the program. Since participating in the initial program, 27% of fathers (*n* = 55) participated in other father-child programs delivered by the research team at the University of Newcastle. For the sub-sample of fathers that completed interviews (*n* = 24), there was an equal distribution of fathers by program year due to the stratification process (4 fathers per year from 2015 to 2020). The sub-sample was comparable to the full survey sample, with most enrolling one daughter (*n* = 21, 87.5%) and 29.2% (*n* = 7) having participated in other similar programs delivered by the research team at the University of Newcastle.

### Demographics and physical activity level of daughters

The mean age of fathers and daughters at the time of survey completion was 47.5 ± 4.9 and 14.4 ± 2.4 years, respectively. Most fathers had a university degree (*n* = 148, 75%), were working in full-time employment (*n* = 184, 93%), and were married (*n* = 174, 88%). Almost all fathers were the biological parent of the daughter they participated with (*n* = 194, 98%), and a small number (*n* = 16, 8%) had changed their relationship status since participating in the program. Of those who had changed their relationship status (*n* = 16), most (*n* = 13, 81%) reported that it had affected the amount of time spent with their daughter, with the majority of these (*n* = 9, 69%) indicating that they now spend less time with her. Demographic characteristics of fathers that undertook interviews were comparable to the total survey sample (Table 2).

**Table 2:** Demographics of total survey sample (*n* = 198) and sub-sample of fathers who completed interviews (*n* = 24)

Variable	Category	Survey sample (*n* = 198)	Sub-sample of fathers who completed interviews (*n* = 24)
Questions about fathers/father figures			
Age (years)	Mean (SD)	47.5 (4.9)	46.9 (4.9)
Highest level of qualification	School certificate (Yr 10 or equiv)	2 (1.0%)	0 (0.0%)
	Higher school certificate (Yr 12 or equiv)	6 (3.0%)	0 (0.0%)
	Trade/Apprentice	10 (5.1%)	0 (0.0%)
	Certificate/Diploma	32 (16.2%)	3 (12.5%)
	University degree	91 (46.0%)	12 (50.0%)
	Higher University Degree	57 (28.8%)	9 (37.5%)
Student status	No	188 (94.9%)	21 (87.5%)
	Yes, part-time student	8 (4.0%)	2 (8.3%)
	Yes, full-time student	2 (1.0%)	1 (4.2%)
Employment status	Full-time paid	184 (92.9%)	21 (87.5%)
	Part-time paid	4 (2.0%)	0 (0.0%)
	Casual paid	1 (0.5%)	1 (4.2%)
	Unemployed	1 (0.5%)	0 (0.0%)
	Retired	2 (1.0%)	1 (4.2%)
	Other	6 (3.0%)	1 (4.2%)
Current relationship status	Single	1 (0.5%)	0 (0.0%)
	In a relationship but not living with partner	2 (1.0%)	0 (0.0%)
	Living with a partner	10 (5.1%)	2 (8.3%)
	Married	174 (87.9%)	21 (87.5%)
	Separated	6 (3.0%)	1 (4.2%)
	Divorced	5 (2.5%)	0 (0.0%)
Change in relationship status since participating in the program	Yes	16 (8.1%)	1 (4.2%)
	No	181 (91.4%)	23 (95.8%)
	I don’t want to answer	1 (0.5%)	0 (0.0%)
Relationship to daughter	Biological father	194 (98.0%)	24 (100.0%)
	Adoptive father	1 (0.5%)	0 (0.0%)
	Step-father	3 (1.5%)	0 (0.0%)
***Questions about Daughters***			
Current age (years)	Mean (SD)	14.4 (2.4)	14.2 (2.9)
Current school year	Year 4	12 (6.1%)	2 (8.3%)
	Year 5	10 (5.1%)	3 (12.5%)
	Year 6	22 (11.1%)	2 (8.3%)
	Year 7	27 (13.6%)	3 (12.5%)
	Year 8	25 (12.6%)	2 (8.3%)
	Year 9	29 (14.6%)	5 (20.8%)
	Year 10	28 (14.1%)	2 (8.3%)
	Year 11	20 (10.1%)	1 (4.2%)
	Year 12	12 (6.1%)	2 (8.3%)
	Graduated high school	13 (6.6%)	2 (8.3%)

Daughters met physical activity recommendations (at least 60 min per day of MVPA) on average 2.8 (± 1.9) days per week. Approximately one-third (*n* = 64, 31.8%) of fathers reported that the COVID-19 pandemic had affected their daughter’s participation in organised sport and/or physical activity. Most of these fathers (86%) indicated that participation had decreased, while 14% described a mixed effect (e.g., reduced team sport participation but increased individual or family-based activities).

### Long-term impacts of the program

From here onward, qualitative themes and sub-themes derived from interviews are reported within the context of the quantitative survey, with quantitative findings interwoven throughout to extend the qualitative insights. These themes relate to the sustained impacts of the program on: (i) daughters, (ii) fathers, (iii) the father-daughter dyad, and (iv) the family unit.

Table 3 provides an overview and summary of all key themes and sub-themes. For each ‘impact on whom’ category, themes are presented below in numerical order based on most often mentioned and strongest impact.

**Table 3:** Overview of themes and sub-themes for long-term impact of the program*

Impact on whom	Theme	Sub-theme
**Daughters**	D1. More sports participation and active lifestyle.	D1a. Increased participation in organised sports.
		D1b. Activity leaders among friends.
		D1c. Willingness to try new sports.
	D2. Enhanced social-emotional wellbeing.	D2a. Resilience and persistence in sport, school and life.
	D3. Awareness and ability to overcome gender inequities.	D3a. Increased knowledge and awareness of gender equity.
		D3b. Gender equity advocates for family and wider community.
**Fathers**	F1. Gender equity advocates.	F1a. Increased knowledge and awareness of gender equity.
		F1b. Gender equity advocates for daughters, family and wider community.
	F2. Prioritising the father role	F2a Increased one-on-one time with daughter.
		F2b. Understanding the important influence they have on their daughters’ lives’.
	F3. Empowering daughters to stay active	F3a. Increased confidence and skills to engage daughters in sport and physical activity.
		F3b. Support daughters in challenges associated with ongoing sport participation.
	F4. More active lifestyle	F4a. Increased awareness of the importance of physical activity.
		F4b. Increased co-physical activity with daughter.
**Father-daughter dyad**	FD1. Increased co-physical activity	FD1a. Prioritising one-on-one time and bonding through co-physical activity.
		FD1b. Practising sports skills.
		FD1c. Continuation of rough & tumble play.
	FD2. Closer relationship in the teenage years.	FD2a. Increased one-on-one time.
		FD2b. Enhanced physical contact.
		FD2c. Deeper and more meaningful conversations.
**Family unit**	Fam1. Gender equity advocates	Fam1a.Increased knowledge and awareness of gender equity.
	Fam2. New family routines	Fam2a. Role models for children.
		Fam2b. Stronger relationships between mother and father.

*For each ‘impact on whom’ category, themes are presented below in numerical order based on most often mentioned and strongest impact

#### Long-term impact of the program on daughters

##### D1. More sports participation and active lifestyle

The most pronounced long-term impact on daughters was increased participation in organised sports. This theme is represented across three sub-themes: D1a. Increased participation in organised sports, D1b. Activity leaders among friends, and D1c. Willingness to try new sports.

- D1a. Increased participation in organised sports
  - Many daughters continued participation in sports introduced during the program or took up new sports that were previously outside their confidence or interest:
    *“She’s played soccer forever*,* but more of the bat and ball sports. She gave them a go*,* yeah*,* whereas before it was like*,* “no*,* I can’t hit a ball with the bat”*,* you know*,* she’d leave that alone.” ID119*,* 5 years post program completion.*
    *“She is now completely immersed in sports. She plays rep netball. She’s played*,* she signed up for cricket this year. She’s doing touch football. So yeah*,* she went from being like a little tiny little princess where she would only wear little ballet shoes everywhere she went.” ID127*,* 7 years post program completion.*
    Survey findings supported these qualitative insights. Within the last 12-months prior to survey completion, daughters participated in an average of 3.6 sports/activities at any level, with the most popular being football/soccer (*n* = 76, 37.8%), swimming (*n* = 73, 36.3%) and walking for exercise (*n* = 71, 35.3%). Further information on the types of sports/activities that daughters participated in with a breakdown of participation level (e.g., home/social, school, community/club sport, regional or state and national or international) is provided in Additional file 2, Supplementary Table 1. In addition, since participating in the program, the average number of sports/activities daughters continued to participate in was 1.9. As a proportion of those who indicated their daughter’s involvement in a specific sport/activity, the most common sports/activities that daughters continued to participate in since program completion were bicycle/scooter riding (total participants = 51, continuers = 38, 74.5%), walking for exercise (total participants = 65, continuers = 43, 66.2%) and swimming (total participants = 104, continuers = 64, 61.5%). Daughters were more likely to discontinue participation in gymnastics (total participants = 39, discontinuers = 21, 53.8%), dancing (total participants = 63, discontinuers = 31, 53.4%) and cricket (total participants = 21, discontinuers = 8, 38.1%). The main reasons for discontinuation were: preferred other sport/activity (*n* = 55, 48.7%), lack of enjoyment (*n* = 44, 38.9%) and time restraints due to schoolwork (*n* = 25, 22.1%). Additional file 2, Supplementary Table 2 provides a comprehensive breakdown of the sports/activities that daughters continued to participate in, newly took up and discontinued since program completion.

- D1b. Activity leaders among friends
  - Several fathers described daughters becoming leaders in physical activity amongst their friendship groups or at school:
    *“That’s actually been a really pleasing thing for us to see - that she’s now the one trying to encourage her friends*,* or she’s bucking the trend*,* because all her friends just want to be cool and just walk it [cross-country] and not do anything*,* but she will actively sort of try and get her friends to run and compete.” ID207*,* 3 years post program completion.*

- D1c. Willingness to try new sports.
  - Some fathers spoke of a greater willingness among daughters to try new sports and activities, particularly those that are traditionally male-dominated:
    *“I think the DADEE thing kind of opened her eyes to sports that are traditionally team-based. Kind of helped her*,* you know*,* transition into those sorts of sports. Soccer first*,* and then AFL [Australian Rules Football]…seeing that she could engage with these traditionally more male-focused ball sports and things like that*,* I think*,* inspired her.” ID67*,* 4 years post program completion.*
    *“The other thing that has been really positive - she plays handball with the boys at school now*,* where definitely she never used to. I remember probably around the time [of the program] she used to say that she didn’t want to run around at lunch because she got sweaty. So that seems to have changed*,* so she’s definitely more physically active than she used to be.” ID29*,* 4 years post program completion.*
    These findings are supported by survey results which showed daughters had taken up an average of 1.7 new sports/activities since participating in the program, with the most common being: fitness/gym (total participants = 63, new to the sport/activity = 47, 74.6%), Oztag [a non-tackling variation of Rugby League] (total participants = 28, new to the sport/activity = 20, 71.4%) and Australian Rules Football/AFL (total participants = 24, new to the sport/activity = 16, 66.7%). Fathers reported the program had played a role in both daughters continued participation in sport (74% selected agree or strongly agree) and the taking up of new sports (58% selected agree or strongly agree).
    This willingness to ‘give things a go’ appeared to be underpinned by improved confidence in the basic skills required for sport or, for some, a new approach to learn or develop sporting skills or performance through step-by-step skill acquisition:
    *“She’ll put her hand up and get involved and I think that’s probably the biggest - it’s like a life skill that*,* like I don’t have to be the best at this*,* but I know I can*,* I could do the basics okay*,* so it doesn’t have to be*,* you know stand back and not get involved*,* cause I’m ashamed that I can’t catch a ball or throw a ball.” ID110*,* 8 years post program completion.*
    Overall, these results are supported by the survey findings, whereby fathers reported that the program had a long-term positive impact on: their daughter’s sports skills (74% selected either agree or strongly agree), their participation in physical activity (74% selected either agree or strongly agree) and their participation in sport (64% selected either agree or strongly agree).

##### D2. Enhanced social-emotional wellbeing

Apart from increased engagement in physical activity, a prominent perceived long-term impact of the program on daughters was improved resilience and willingness to persevere in the face of challenges, which extended beyond sport. This is reflected in the following sub-theme.

- D2a. Resilience and persistence in sport, school and life
  - Fathers frequently described daughters displaying a more proactive attitude to improve performance in both academic and sporting ventures, in which they were perceived to display a greater sense of resilience in the face of ‘failure’ and take responsibility for improving through practice:
    *“I think she became a lot more persistent at studying. She really focused on becoming better at maths and English and science*,* and that she started to see really good results from that*,* from applying herself and being persistent*,* and she’s even carried that over again even further into high school now.” ID164*,* 5 years post program completion.*
    *“Just recently she played in a final of football and had the opportunity to kick probably a winning goal and missed and that could be very disappointing for someone*,* and I was disappointed for her. But the positive out of it is that she was like*,* “Well*,* you know*,* that’s a skill error. I’ll go and practice.” ID67*,* 4 years post program completion.*
    Program participation was also seen to be linked to an increased interpersonal resilience and confidence which had led to some daughters displaying what some fathers referred to as “toughness”, “bravery” and leadership skills;
    *“She went from being a passive person within her friends’ group to leading that friends’ group after the program.” ID72*,* 6 years post program completion.*
    Tied in with the increased level of physical activity experimentation, which many had witnessed within various sports and activities, fathers similarly spoke of a certain level of confidence to give things a go which was seen to stem from increased resilience or acceptance of challenges;
    *“I think physically and emotionally there’s been a huge change and evolution for [daughter name] and I don’t think she would be where she is with that confidence and that resilience and that leadership without attending the DADEE program and experiencing that.” ID72*,* 6 years post program completion.*
    *“She comes home from school and says like*,* “Oh*,* I’ve joined the touch footy team” and I’m like*,* “Have you ever played touch footy before?” And she’s like*,* “Nup*,* but I’ll pick it up.” And off she goes and plays touch footy.” ID67*,* 4 years post program completion.*
    These findings are supported by the survey results, with fathers reporting that the program had a long-term positive impact on their daughter’s social-emotional wellbeing (75% selected either agree or strongly agree).

##### D3. Awareness and ability to overcome gender inequities

Several fathers acknowledged the program as an important catalyst for their daughter’s awareness of gender equity and empowerment to challenge stereotypes. This theme is represented across two sub-themes D3a. Increased knowledge and awareness of gender equity, and D3b. Gender equity advocates for family and wider community.

- D3a. Increased knowledge and awareness of gender equity
  - Fathers described daughters becoming more confident in asserting themselves and recognising inequities:
    *“The confidence that she’s got with herself now*,* it allows her to make a stance on what she sees is acceptable and what’s not basically acceptable*,* and I think that shy little young girl that has changed and evolved so much*,* and learnt so much from that experience. And that’s the long*,* lasting impression that this program has had*,* and I think that’s quite positive.” ID72*,* 6 years post program completion.*

- D3b. Gender equity advocates for family and wider community
  - Many daughters were described as actively identifying gender inequities, vocally addressing and changing these within their family and wider community:
    *“She came home last week and said her teacher came into the room and said*,* “I’m after two strong boys to help me lift all the tables in the other room.” And she put her hand up and she was like*,* “I am stronger than any of the boys.” ID133*,* 4 years post program completion.*
    These qualitative results are confirmed by the quantitative survey results with fathers reporting that the program had a long-term impact on their daughter’s awareness of gender equity issues (80% selected either agree or strongly agree).

#### Long-term impact of the program on fathers

##### F1. Gender equity advocates

The greatest long-term impact for fathers was increased awareness and communication around gender bias. This is presented across two sub-themes: F1a. Increased knowledge and awareness of gender equity and F1b. Gender equity advocates for daughter, family and community.

- F1a. Increased knowledge and awareness of gender equity
  - Many fathers described eye-opening experiences regarding societal gender bias:
    *“I was like*,* “Surely that’s not the case.” And then I went away and did my own research and it’s like*,* “Wow.” Sort of blows your mind when you actually look. It’s no wonder why girls aren’t motivated to stick with things because they are sort of put in a box. So*,* I guess*,* that’s something that all four of us definitely don’t accept…It was like that was definitely a lightbulb moment and that stuck with me. You know what I mean? That sort of definitely had a lasting effect. I would say…I think it sort of affects your whole parenting as well”. ID133*,* 4 years post-program completion.*
    *“[It] definitely did make me stop and think*,* how I’m going to refer to a group of people…. Since that program sort of it definitely did open my eyes up to the gender stereotype stuff. We did make sure that the last Olympic games. We made sure we were putting on a lot of the female sports”. ID107*,* 5 years post-program completion.*

- F1b. Gender equity advocates for daughter, family and community
  - The knowledge around these biases were reported to have had a sustained and powerful impact on father’s communication; not just with their daughters and immediate family, but in their broader sphere of influence, such as their workplaces:
    *“It was rewarding educationally and certainly got me thinking of those gender stereotype issues and female empowerment. So*,* I think it was a good thing*,* and it probably opened up our lines of communication*,* and actually talking about those issues which I probably wouldn’t have talked with her about.” ID26*,* 8 years post-program completion.*
    *“I’ve been involved with the Diversity Inclusion Committee here at work for a little while*,* too. And I’ve been really conscious of this since the DADEE program and more so now at work.” ID107*,* 5 years post-program completion.*
    These findings align with survey results, with fathers reporting that the program had a long-term impact on their own awareness of gender equity issues (82% selected either agree or strongly agree).

##### F2. Prioritising the father role

A key long-term impact of the program for fathers was a strengthened sense of identity and purpose in their parenting role. This is reflected across the sub-themes F2a. Increased one-on-one time with daughter and F2b. Understanding the important influence they have on their daughters’ lives.

- F2a. Increased one-on-one time with daughter
  - For many participants, the program had brought about an important shift in prioritising time with their daughter and family at large and, in particular, one-on-one time, which often had meant a shift away from traditional ‘breadwinner’ attitudes:
    *“Hey*,* your career is the most important thing and that means that comes with the sacrifice of the family. That means that the mother has to have the relationship with the daughter or blah blah blah”; it’s like*,* her and I [are] able to have a really close relationship together and whatever*,* just trying to think of how to put this*,* but I think that program really [made a] significant contribution to making that actually real*,* actually like*,* ‘hey*,* yeah*,* you can make this happen’.” ID31*,* 6 years post program completion.*
  F2b. Understanding the important influence they have on their daughters’ lives
  - This re-prioritisation also came with a re-evaluated or newly developed sense of the important role that these fathers had in their daughters’ lives:
    *“I think it’s just that elevation of consciousness around my role as a dad*,* particularly with daughters*,* and how pivotal that is in their lives*,* I guess sort of*,* being conscious of that.” ID110*,* 8 years post program completion.*
    These findings are consistent with survey results, with fathers reporting that the program had a positive long-term impact on their ‘role as a father’ (87% selected either agree or strongly agree) and their parenting practices (86% selected either agree or strongly agree).

##### F3. Empowering daughters to stay active

Fathers described feeling better equipped to encourage their daughters’ participation in sport and physical activity, reflected in two sub-themes: F3a. Increased confidence and skills to engage daughters in sport and physical activity, and F3b. Support daughters in challenges associated with ongoing sport participation.

- F3a. Increased confidence and skills to engage daughters in sport and physical activity
  - Many fathers talked of the confidence and skills they had gained to teach their daughters new sport skills and also support their daughters in facing challenges and staying physically active:
    *“Me knowing*,* or having an idea of*,* how to work with her on that to get her into another sport that is a completely different skillset to what she normally does; and for her to be able to do that and then really enjoy it. Now if I hadn’t done the program*,* I would’ve given up*,* she would’ve given up.” ID28*,* 7 years post program completion.*
    The fathers had, in particular, found the statistics presented around girls’ sports participation into and beyond the teenage years confronting and eye-opening and this had, for many, led to a personal dedication to consciously promote and encourage their daughters to remain active and involved in sports:
    *“The single long-lasting effect is I’ve just got to support my daughter through the next*,* whatever it is*,* 6 years to encourage her to keep going when things*,* yeah*,* when she may not want to for various reasons and it’s easier to opt out.” ID29*,* 4 years post program completion.*

- F3b. Support daughters in challenges associated with ongoing sport participation
  - Some fathers were already experiencing waning interest in sports participation among their teenage daughters and acknowledged the presence of strong external influences of peers responsible for this. The knowledge gained from the program was therefore, for many, playing an important role in strengthening their long-term commitment to keeping their daughters’ interest in sport and physical activity alive:
    *“Given my daughter’s now at that age where she’s 12. We are having discussions about her not wanting to do certain sports*,* and I guess it’s probably made me a bit more resolute that just*,* without sounding too pushy*,* I’ve got to be stronger around*,* ‘You have to keep doing sport.’” ID29*,* 4 years post program completion.*

##### F4. More active lifestyle

Fathers described the program as positively impacting their own activity levels, reflected across two sub-themes: F4a. Increased awareness of importance of physical activity, and F4b. Increased co-physical activity with daughters.

- F4a. Increased awareness of the importance of physical activity
  - Many fathers said the program had acted as a catalyst for change, or at least a shift, in the importance they placed on a healthy lifestyle:
    *“I think it is one of the early catalyst sorts of things that happened that made me be more aware of my own fitness*,* and to take responsibility for that as well.” ID31*,* 6 years post program completion.*

- F4b. Increased co-physical activity with daughter
  - There was a strong sense that the desire to stay physically fit and capable was grounded in a desire to stay actively involved with their daughters:
    *“My physical activity has sort of increased exponentially too*,* from when I look back to when I did that program*,* I’m doing heaps more sport now than what I’ve ever done*,* spending lots more time with the kids outdoors*,* bike riding on the weekends*,* those sorts of things. It’s had a massive impact and I’ve got a boy and a girl.” ID208*,* 3 years post program completion.*
    These findings are consistent with survey results, with fathers reporting that the program had a positive long-term impact on their physical activity (67% selected either agree or strongly agree).

#### Long-term impact of the program on the father-daughter dyad

##### FD1. Increased co-physical activity

Fathers commonly described sustaining shared physical activity with their daughters well beyond program completion. This is reflected across three sub-themes: FD1a. Prioritising one-on-one time, FD1b. Practising sports skills, and FD1c. Continuation of rough and tumble play.

- FD1a. Prioritising one-on-one time and bonding
  - Being proactive in seeking out physically active time together and an actual increase in the time spent together were prominent impacts of the program on the father-daughter relationship. Many fathers spoke of a more conscious effort in seeking those bonding moments, which were now more likely to revolve around being active together:
    *“Yeah*,* just doing activity together*,* getting out of the house*,* getting away from the ipads*,* just that sort of sports stuff*,* because she thrives on it*,* and just enjoying a bit more outdoor play…” ID208*,* 3 years post program completion.*

- FD1b. Practising sports skills
  - For some, time together was an opportunity for fun and bonding, whereas for others this was a time to learn and practice skills:
    *“[It] made me more aware of these basic skills that the girls’ sort of have when they’re young that they then lose if they didn’t practice them*,* was kind of part of my role is to help increase their skills - mainly to give them confidence to stay involved in sport…we quite regularly go and do things like*,* go down the park and throw a tennis ball to each other and just practice our throwing skills and catching.” ID110*,* 8 years post program completion.*

- FD1c. Continuation of rough and tumble play
  - For many, the benefits of the rough and tumble play that they learned from the program lived on as daughters got older:
    *“She still wants to wrestle with me. Yeah*,* so that bond between myself and her. It was pretty good before the program. But yeah*,* ever since then she’s hasn’t been afraid to …. have a bit of a wrestle.” ID107*,* 5 years post program completion.*
    While a number of fathers spoke of the difficulties and barriers of a busy family and work life getting in the way of active time together with their daughters, there was evidence of a clear shift in the focus and importance placed on such time:
    *“I definitely haven’t done as much as I thought I would have for the one-on-one playing games*,* training with them*,* practising with them. But I guess what the difference is I’m actually thinking about it*,* whereas before Dads and Daughters I would have thought nothing of it*,* I would have thought nothing of pretty much ignoring the fact that it would probably be a good thing to go out and now go for a run or kick a ball around with my girls.” ID119*,* 5 years post program completion.*
    These findings are supported by survey results. Fathers reported they were participating in co-physical activity with their daughter and other family members for an average of 1.5 (± 1.6) days/week. Co-physical activity, one-on-one with their daughter, was slightly lower, with an average of 1.2 (± 1.6) days/week.

##### FD2. Closer relationship in the teenage years

Many fathers felt that the program had strengthened their connections with daughters as they entered adolescence, reflected across three sub-themes: FD2a. Increased one-on-one time, FD2b. Enhanced physical contact, and FD2c. Deeper conversations.

- FD2a. Increased one-on-one time
  - Many talked of an increased ‘presence’ in father-daughter interactions where both were found to actively seek out these one-on-one opportunities:
    *“She’s more willing to have that engagement - that one-on-one engagement. She’ll come to watch the other kids do swimming lessons at the moment*,* and she wants that because she gets to have that one-on-one time with me. We get to sit in the stands*,* and chat and talk about what’s going on with school and things*,* where maybe she wouldn’t basically want to have that interaction with me if she hadn’t done this program.” ID72*,* 6 years post program completion.*

- FD2b. Enhanced physical contact
  - Another change to the dynamic, mentioned by some fathers, involved a greater degree of comfort with physical contact:
    *“Still*,* from that program as well*,* she always used to then wanna come up and give a firm cuddle and stuff*,* and so she’ll still do that now*,* like go to school and home and it just became a natural thing.” ID113*,* 8 years post program completion.*

- FD2c. Deeper and more meaningful conversations
  - Others talked of having deeper and more meaningful conversations with their daughters than they felt they otherwise would have, with some fathers finding themselves becoming a confidante for their daughter:
    *“We’ve got a super connection where she’ll literally tell me anything…we tell each other everything. Everything’s open and honest…I remember at the end of the program…. we both wrote really open and honest letters. And I think from that point on*,* that sort of helped open the lines of communication I reckon.” ID133*,* 4 years post program completion.*
    These findings are consistent with survey results, with fathers reporting that the program had a long-term positive impact on the relationship with their daughter (89% selected either agree or strongly agree).

#### Long-term impact of the program on the family unit

Almost all participants reflected on a flow-on effect from the program to the broader family unit. This involved their partners and other children benefitting from the insights learnt in the program and sharing in new experiences and activities that had become part of the family routine:

*“I think the benefits flowed onto the other girls as well. Whilst they were 2 years older - they would have been 14*,* it’s still a very influential time in their life.” ID26*,* 8 years post program completion.*

##### Fam1. Gender equity advocates

While most participants did voice having had pre-existing family dynamics and attitudes aligned with gender equity, the family unit impact was, in many cases, discussed in relation to a new focus and emphasis on gender awareness. This is reflected in the following sub-theme.

- Fam1a. Increased knowledge and awareness of gender equity
  - This took the form of an intentional celebration of women’s sport (e.g., watching women’s sport together), ‘calling out’ instances of ‘pinkification’ used in shops and marketing, and generally leading to an increased openness about gender equity, over and above their more ‘hidden’ pre-existing attitudes. Alongside this more prominent gender equity advocacy, a spotlight on women’s sports participation was evident on a family wide level:
    *“Learning and realising those facts and figures that I was talking about before has certainly been*,* you know*,* it resets your outlook. So*,* your whole perspective*,* the way you look at bringing up daughters changes from that moment that you get that knowledge so that long*,* lasting impact*,* which has really encouraged me to take the girls to women’s sport in particular*,* and to encourage them to watch and to get involved with Women’s Sport*,* particularly the Women’s Football and Rugby League… I wouldn’t have done as much if I didn’t go to that program.” ID123*,* 8 years post program completion.*

##### Fam2. New family routines

In some cases, program participation had been a catalyst for different family routines and priorities around how time was spent and in some instances both mother and father had found new ways to bond and be role models for their children. This is reflected in the two sub-themes: Fam2a. Role models for children and Fam2b. Stronger relationships between mother and father.

- Fam2a. Role models for children
  - Fathers described adopting new habits and modelling active lifestyles:
    *“It’s had an impact on just myself*,* obviously*,* in all the physical activity I do. Obviously*,* my wife’s a bit now the same*,* and we’re probably doing more sort of physical activity together over the last 2 years than we have ever done*,* and so just to give the kids that and then the kids sort of feed off that as well.” ID208*,* 3 years post program completion.*

- Fam2b. Stronger relationships between mother and father
  - Several participants indeed spoke of a stronger relationship with their partner via shared interest and participation in sports or leisure-time exercise:
    *“I think that’s even improved the relationship between my wife and I that we’re able to gym together or go for a walk together…and so we have been looking at trying to do some sport things together.” ID31*,* 6 years post program completion.*
    The one father interviewed who was living in a non-nuclear family situation (divorced and co-parenting children) similarly spoke of some positive outcomes from the program participation in terms of parental relationship, as well as the flow-on effect on the child taking part. This suggests a potential protective effect of the program on children living in complex family situations:
    *“I had separated and was divorced basically from her mother at that time*,* so there was an opportunity to spend more time with [daughter name] and provide that one-on-one. And I believe that had a really positive influence on the ongoing external issues as well. I think it actually helped basically both things because it meant more interaction and more*,* basically*,* communication and then the fact that [daughters name]’s mum actually came to one of the sessions when the mums were invited as well*,* I think was a successful outcome for helping mend a few things as well.” ID72*,* 6 years post program completion.*

## Discussion

The current study aimed to investigate the long-term impact of the ‘Dads and Daughters Exercising and Empowered’ program on the fathers, daughters and broader family unit when followed up between 3–8-years post-program. To our knowledge, this is the longest follow-up time period of any parenting intervention that has targeted child physical activity and/or wellbeing. Findings confirmed several long-term impacts on daughters, fathers, and the wider family unit. For daughters, three key themes emerged as prominent sustained impacts: *D1. More sports participation and active lifestyle*, *D2. Enhanced social-emotional wellbeing* and *D3. Awareness and ability to overcome gender inequities.* For fathers, four key themes emerged: *F1. Gender equity advocates*,* F2. Prioritising the father role*,* F3. Empowering daughters to stay active* and *F4. More active lifestyle*. Two key themes emerged for the long-term impacts on the father-daughter dyad: *FD1. Increased co-physical activity* and *FD2*. *Closer relationship in the teenage years*, while two themes also emerged for the long-term impacts on the wider family unit: *Fam1. Gender equity advocates*, and *Fam2. New family routines.*

There are several reasons pertaining to how and why this 9-week program may have had such a profound impact when followed up between 3 and 8 years after program completion. More generally, the theoretical underpinnings of the program were based upon key constructs of Self-Determination Theory (i.e., autonomy, competence, relatedness) and Social Cognitive Theory (e.g., self-efficacy, goals, social support), which have been associated with long-term behaviour change [47–49]. Due to the novelty of the dataset and the lack of long-term follow-up in similar interventions, comparisons with other studies are challenging. However, a more specific exploration into the potential reasoning for the unique long-term impacts across daughters, fathers, and the wider family unit is provided below.

### Potential reasoning for the long-term impact of the program on daughters

In daughters, the strongest impact related to *D1. More sports participation and active lifestyle*. This finding is important given that many of the girls within the sample have entered the teenage years (mean age 14.4 ± 2.4 years) and there is a notable decline in physical activity observed in girls during adolescence [50]. Between three and eight years after completing the program, daughters were meeting physical activity recommendations on an average of 2.8 (± 1.9) days/week. This represents a 6.7% decline when compared with a previous non-randomised pre-post study at 12-month follow-up (3.0 ± 1.5 days/week) and a 15.2% decline compared with post-program assessments (3.3 ± 1.6 days/week) [15]. As inferential analyses could not be conducted in the present study, these descriptive comparisons should be interpreted cautiously, as they reflect differences in study design rather than statistical comparison. Despite this, it is important to consider these results, both in the context of the well-established declining levels of MVPA among girls, and the global health promotion goal of reducing this decline [51]. Longitudinal research shows on average, a 5.3% (95% CI, − 7.6 to − 3.0) decline per year in MVPA among girls with the decline more pronounced at age 13 (− 8.4%, 95% CI, − 13.2 to − 3.7) [52], which is close to the mean age of the sample in this study. There are also the effects of the COVID-19 pandemic and subsequent strict lockdowns, with 32% of fathers in the present study reporting that the COVID-19 pandemic had impacted on their daughters’ participation in organised sport and/or physical activity, with most of these (86%) confirming it had reduced their participation. This coincides with the state-wide impact of COVID-19 in NSW (Australia), with 70% of 16,177 parents surveyed confirming that since COVID-19 restrictions, their children’s physical activity levels had decreased (either a little or a lot), with the decrease most notable amongst adolescents aged 12 years or over [53]. Furthermore, the same study found that, post-COVID, fewer females than males returned to playing community sport [53]. Considering all of these factors, the physical activity levels reported by daughters in this study indicate a reduction in the expected decline of physical activity among girls. In addition, fathers in the current study, reported that daughters participated in an average of 3.6 sports/activities at any level, with the most popular being football/soccer, swimming, and walking for exercise, while daughters also took up an average of 1.7 new sports/activities since participating in the program with the most common being: fitness/gym (total participants = 63, new participants = 47, 74.6%), Oztag (total participants = 28, new participants = 20, 71.4%) and Australian Rules/AFL (total participants = 24, new participants = 16, 66.7%). This active lifestyle by daughters, is likely attributed to the program’s focus on:

1. Fundamental movement skill (FMS) proficiency: daughters were taught to become proficient in FMS for ball sports: kicking, catching, striking, bouncing, and throwing. This emphasis likely enabled girls to participate more confidently and competently across a wider variety of sports (e.g., mastering ‘kick’ enables daughters to play soccer, AFL, rugby league etc.). FMS competency provides a foundation for an active lifestyle and is strongly associated with lifelong physical activity [54].
2. Psychological resources and skills: daughters had the opportunity to develop psychological resources and skills (e.g., persistence, resilience, bravery, self-reliance etc.). These likely helped them persist in practice and overcome mistakes, facilitating continued participation in sports and physical activity in the context of declining participation amongst their female peers.
3. Father involvement: fathers served as ‘one-on-one’ coaches to their daughters during the program and learnt appropriate pedagogical and parenting skills. These skills likely led to fathers initiating engaging practical activities for their daughters beyond the program. This also links with the father sub-theme: ‘*F3a Increased confidence and skills to engage daughter in sport and physical activity’.* In addition, it has been well-documented that fathers are major influencers on the physical activity and sports participation of their children [55].
4. Breaking down gender stereotypes: changing perceptions of gender-stereotyped attitudes in sport opened more opportunities to practices and try new sports, and positively impacted on physical activity levels [56].

Other prominent themes for long-term impact on daughters were: *D2. Enhanced social-emotional wellbeing* and *D3. Awareness and ability to overcome gender inequities.* Potential reasons for this include the program’s application of an evidence-based pedagogical approach [57] to provide daughters and fathers with accessible, ‘real-life’ practical tips to maximise learning and knowledge retention. Each week, daughters were taught essential social-emotional constructs: self-control, persistence, resilience, kindness, bravery, positivity, critical thinking, and self-reliance. These constructs, known as ‘Words of Empowerment’, were accompanied by catchphrases and actionable steps to help daughters understand the concepts. The focus was on practical tips applicable in both sporting contexts and everyday life. In addition, daughters were taught important critical thinking skills to identify, navigate and challenge gender prejudice that infiltrates all aspects of their lives, particularly in the sporting and physical activity domains. Daughters were also asked to keep diaries between sessions of any gender bias they saw or heard. They were encouraged to discuss these instances with their dad, learning how to address them while developing the understanding that their opportunities in life were not limited by their gender.

### Potential reasoning for the long-term impact of the program on fathers

In fathers, the key themes were similar to the daughters’, but the strongest impact related to *F1. Gender equity advocates*, followed by *F2. Prioritising the father role*,* F3. Empowering daughters to stay active* and *F4. More active lifestyle*. These long-term impacts are likely a result of attending the sessions together with their daughter and the program’s focus on ‘reciprocal reinforcement’ whereby fathers were encouraged to be positive role models and gender equity advocates for their daughters. The daughters were also responsible in driving many home-based activities and encouraged to motivate their fathers to be more active. The overlap in key themes between fathers and daughters may relate to fathers’ unique influence on their daughters, especially in the physical domain [58]. A previous systematic review has shown a modest, positive bi-directional association between father and child physical activity levels [59]. The long-term impact relating to *F2. Prioritising the father role* is important, as research based on observational data indicates that positive father involvement is associated with various favourable developmental outcomes for girls. These outcomes include enhanced self-esteem [60], greater self-reliance, improved academic achievement [61], stronger peer relationships [62], and positive body image [63]. Given the interlinked nature in the long-term impacts between fathers and daughters, this finding may have contributed towards the prominence of *D2. Enhanced social-emotional wellbeing* and *D3. Awareness and ability to overcome gender inequities* for daughters.

### Potential reasoning for the long-term impact of the program on the father-daughter dyad

Within the father-daughter dyad, the strongest impact related to *FD1. Increase in co-physical activity* with sub-themes relating to one-on-one time, practising sport skills and continuing rough and tumble play. This theme was affirmed by survey data which showed a continuation of co-physical activity with their daughter on 1.2 (± 1.6) days/week. It is likely that the continuation of co-physical activity had a direct influence on the second key theme: *FD2. Closer relationship in the teenage years*. Shared physical activity experiences play an essential role in fostering the father-child relationship. Sociological research characterises this bond as an “activation relationship”, developed through co-physical activities and stimulating, unpredictable, wrestling-style play [64, 65]. In a previous qualitative study, both fathers and adult daughters identified joint participation in sporting activities as a pivotal turning point in their relationship during childhood [66]. Importantly, a positive father-child bond is associated with various favourable outcomes across physical, social-emotional, educational and psychological domains for children [67]. Thus, increasing paternal physical activity levels holds great potential for enhancing children’s well-being. Potential reasoning as to why *FD1. Increase in co-physical activity* and *FD2. Closer relationship in the teenage years* were long-lasting may be a result of providing fathers and daughters with highly engaging and enjoyable co-physical activity experiences during the program sessions and at home with a range of challenges. Both were also provided with tips and strategies to modify, adapt and add ‘hooks’ to continually evolve and think of new variations for these activities. Additionally, the program educated fathers on using an authoritative parenting style, characterised by a balance of high parental control and autonomy-supportive behaviours, including warmth, positive communication, and receptiveness [68]. This parenting style is associated with greater engagement in physical activity in children [69] and may explain *FD1. Increase in co-physical activity* and *D1. More sports participation and active lifestyle* among daughters. Furthermore, during the education sessions, fathers were provided with strategies to become more approachable, authentic, responsive and empathetic toward their daughters. This may further explain the impact relating to *FD2. Closer relationship in the teenage years* as, in a study where adult daughters were asked to describe their fathers, those with close relationships were more likely to highlight the aforementioned qualities [70].

### Potential reasoning for the long-term impact of the program on the wider family unit

*Among the wider family unit, the strongest long-term impacts included Fam1. Gender equity advocates*, and *Fam2. New family routines.* It is likely that fathers and daughters who embraced the program concepts may have carried these into the home environment, affecting other family members. Then, each family member influenced the others reciprocally, directly, and indirectly [55]. This aligns with Family Systems Theory, which views the family as a complex and interactive social system where all family members’ needs and experiences affect the others [71].

### Future recommendations

This study builds on the previously established benefits of the ‘Dads and Daughters Exercising and Empowered’ program and highlights the sustained impacts among fathers and daughters, as well as broader benefits to the family unit. Future research will look to optimise the program for large-scale delivery, including mapping of implementation features, engaging key stakeholders, and addressing potential barriers to effective implementation and scale-up. This will be guided by an appropriate implementation framework such as PRACTIS guide [72] or the Intervention Scalability Assessment Tool (ISAT) [73].

### Strengths and limitations

The current study was strengthened by long-term participant follow-up (between 3 and 8 years post-program completion) and the use of both quantitative and qualitative data to enable a broad range of views to be canvassed. In addition, the qualitative analysis was conducted by an independent researcher with considerable expertise in qualitative methods to enhance the credibility of the data. Limitations include an over-representation of fathers who were married, had higher education levels and were in full-time employment, which may limit the generalisability of findings. In addition, for both qualitative and quantitative data, only the perspectives from the fathers were gathered rather than from daughters or the wider family unit.

## Conclusions

This paper provides novel, important evidence on sustained effects (between 3 and 8 years since program completion) of a father-daughter physical activity and wellbeing program (‘Dads and Daughters Exercising and Empowered’). Notably, the long-term effects were established among program participants (i.e., fathers and daughters) but also among the wider family unit. The evidence-based strategies implemented in the program can inform design and delivery of more effective father-daughter lifestyle programs, with potential to achieve long-term, holistic benefits, including increasing physical activity and sports participation, prioritising the father role, strengthening the father-daughter bond, improving social-emotional wellbeing, and advancing gender equity.

## Supplementary Information

Supplementary Material 1.

Supplementary Material 2.

## Data Availability

The de-identified data are available from PJM upon reasonable request.

## Abbreviations

DADEE: Dads and Daughters Exercising and Empowered
FMS: Fundamental Movement Skills
MVPA: Moderate-to-Vigorous Physical Activity
COVID-19: Coronavirus
RCT: Randomised Controlled Trial
