# Supplementary material for: Long-term outcomes of a father-daughter program for sport participation, activity, wellbeing, and gender equity: a 3-8-year mixed-methods follow-up
Source: BMC Public Health. 2026 Jan 30;26:742. doi: 10.1186/s12889-026-26287-7 (PMC12934063; doi:10.1186/s12889-026-26287-7)
Supplement: Supplementary file 1 — Supplementary Material 1. [file 12889_2026_26287_MOESM1_ESM.docx]

Additional File 1

**Long-term outcomes of a father-daughter program for sport participation, activity, wellbeing, and gender equity: a 3-8-year mixed-methods follow-up.**

Philip J. Morgan^1,2^*, Jaqueline A Grounds^1,2^, Rosslyn O’Connor^3^, Daniel R Lee^1,2^, Lee M Ashton^1,2^.

***** Correspondence: Philip.morgan@newcastle.edu.au; Tel.: +61-2-49-217265

**Semi-structured interview guide questions for fathers**

1. Can you tell me when you first did the Daughters and Dads program and where the program was delivered?
   1. Did you do the program with one or more daughters? How old were they at the time you did the program?
2. At the time you did the program, did it have any impact on you, your daughter(s) and/or your family? If yes, what was the impact?

The next questions will ask about any **long-lasting impact** on you, your daughter(s) and your family, from the time you completed the program [insert ‘year’ at ‘location’ from Q1] up to now:

1. What is the greatest long-lasting impact of the program? This could be on you, your daughter and/or your family.

Now I will be asking questions about **[insert daughter(s) name(s)]** that you did the program with:

1. What is the biggest change you continue to see in [insert daughters name] as a result of the program?
2. What long-lasting impact has the program had on [insert daughters name] confidence, resilience, positivity etc? Can you provide examples?
3. Has the program had a long-lasting impact on [insert daughters name] physical activity (e.g. continued sport or taken up a new sport etc.)? Can you provide examples?
4. What long-lasting impact has the program had on [insert daughters name] own awareness of gender stereotypes? Can you give us any examples?

**These questions are about you:**

1. Has the program had any long-term changes on your physical activity, parenting, 1 v 1 time with your daughter? If so, what was the biggest change?
2. What impact has the program had on your ability to continue to engage in physical activity with [insert daughters name]?
3. During the program you and [insert daughters name] learnt about wearing ‘gender glasses’ as way to identify and deal with gender inequity issues. Has this had any long-lasting effects on what you do, how you talk to [insert daughters name] or how you think about gender issues?

**This question is about your relationship with [insert daughters name]:**

1. Since you finished the program, do you feel that is has made any changes to the relationship you have with [insert daughters name]?
2. Is there anything else you would like to add regarding the long-lasting impact of the program?
