# Supplementary material for: Long-term outcomes of a father-daughter program for sport participation, activity, wellbeing, and gender equity: a 3-8-year mixed-methods follow-up
Source: BMC Public Health. 2026 Jan 30;26:742. doi: 10.1186/s12889-026-26287-7 (PMC12934063; doi:10.1186/s12889-026-26287-7)
Supplement: Supplementary file 2 — Supplementary Material 2. [file 12889_2026_26287_MOESM2_ESM.docx]

Additional File 2

**Long-term outcomes of a father-daughter program for sport participation, activity, wellbeing, and gender equity: a 3-8-year mixed-methods follow-up.**

Philip J. Morgan^1,2^*, Jaqueline A Grounds^1,2^, Rosslyn O’Connor^3^, Daniel R Lee^1,2^, Lee M Ashton^1,2^.

***** Correspondence: Philip.morgan@newcastle.edu.au; Tel.: +61-2-49-217265

**Table of Contents**

| **Supporting information item** | **Page** |
| --- | --- |
| **Supplementary Table 1.** Daughters’ participation level for sport and physical activities in the last 12-months. | 2 |
| **Supplementary Table 2.** Sports/activities for which daughters continued to participate in, newly took up and dropped out of since participating in the program. | 3 |

**Supplementary Table 1:** Daughters’ participation level for sport and physical activities in the last 12-months *

| **Sport or physical activity** | **Home / local park / social**  **with friends and family** | **School -based** | **Community/club sport** | **Regional or state representative** | **National or International representative** |
| --- | --- | --- | --- | --- | --- |
| Football/ soccer (n=76) | 4 (5.3%) | 7 (9.2%) | 60 (78.9%) | 5 (6.6%) | 0 (0.0%) |
| Swimming (n=73) | 24 (32.9%) | 14 (19.2%) | 29 (39.7%) | 5 (6.8%) | 1 (1.4%) |
| Walking for exercise (n=71) | 64 (90.1%) | 6 (8.5%) | 1 (1.4%) | 0 (0.0%) | 0 (0.0%) |
| Netball (n=62) | 3 (4.8%) | 10 (16.1%) | 45 (72.6%) | 4 (6.5%) | 0 (0.0%) |
| Fitness/ gym (n=58) | 34 (58.6%) | 7 (12.1%) | 17 (29.3%) | 0 (0.0%) | 0 (0.0%) |
| Running/jogging (n=56) | 32 (57.1%) | 12 (21.4%) | 4 (7.1%) | 7 (12.5%) | 1 (1.8%) |
| Bicycle/scooter riding (n=47) | 45 (95.7%) | 0 (0.0%) | 1 (2.1%) | 1 (2.1%) | 0 (0.0%) |
| Touch football (n=39) | 2 (5.1%) | 7 (17.9%) | 28 (71.8%) | 2 (5.1%) | 0 (0.0%) |
| Dancing (n=35) | 2 (5.7%) | 9 (25.7%) | 18 (51.4%) | 4 (11.4%) | 2 (5.7%) |
| Basketball (n=31) | 4 (12.9%) | 9 (29.0%) | 16 (51.6%) | 1 (3.2%) | 1 (3.2%) |
| Athletics (n=26) | 2 (7.7%) | 14 (53.8%) | 5 (19.2%) | 4 (15.4%) | 1 (3.8%) |
| Tennis (n=24) | 8 (33.3%) | 3 (12.5%) | 12 (50.0%) | 1 (4.2%) | 0 (0.0%) |
| Oztag (n=21) | 0 (0.0%) | 5 (23.8%) | 14 (66.7%) | 2 (9.5%) | 0 (0.0%) |
| Australian Rules / AFL (n=20) | 0 (0.0%) | 6 (30.0%) | 12 (60.0%) | 2 (10.0%) | 0 (0.0%) |
| Gymnastics (n=20) | 1 (5.0%) | 5 (25.0%) | 13 (65.0%) | 1 (5.0%) | 0 (0.0%) |
| Cricket (n=16) | 1 (6.3%) | 3 (18.8%) | 10 (62.5%) | 2 (12.5%) | 0 (0.0%) |
| Yoga/pilates (n=11) | 8 (72.7%) | 0 (0.0%) | 3 (27.3%) | 0 (0.0%) | 0 (0.0%) |
| Volleyball (n=3) | 0 (0.0%) | 2 (66.7%) | 1 (33.3%) | 0 (0.0%) | 0 (0.0%) |
| Rugby (n=2) | 0 (0.0%) | 0 (0.0%) | 2 (100.0%) | 0 (0.0%) | 0 (0.0%) |
| Other (n=42) | 8 (19.0%) | 4 (9.5%) | 24 (57.1%) | 3 (7.1%) | 3 (7.1%) |

*Multiple responses could be selected

**Supplementary Table 2:** Sports/activities for which daughters continued to participate in, newly took up and dropped out of since participating in the program.*

| **Sport or physical activity** | **Continued to participate in since participating in the program** | **Taken up new participation since participating in the program** | **Dropped out since participating in the program** |
| --- | --- | --- | --- |
| Swimming (n=104) | 64 (61.5%) | 5 (4.8%) | 36 (34.6%) |
| Football/ soccer (n=96) | 42 (43.8%) | 31 (32.3%) | 29 (30.2%) |
| Netball (n=78) | 34 (43.6%) | 28 (35.9%) | 18 (23.1%) |
| Walking for exercise (n=65) | 43 (66.2%) | 22 (33.8%) | 2 (3.1%) |
| Fitness/ gym (n=63) | 14 (22.2%) | 47 (74.6%) | 3 (4.8%) |
| Dancing (n=58) | 24 (41.4%) | 7 (12.1%) | 31 (53.4%) |
| Running/jogging (n=54) | 29 (53.7%) | 24 (44.4%) | 2 (3.7%) |
| Bicycle/scooter riding (n=51) | 38 (74.5%) | 9 (17.6%) | 4 (7.8%) |
| Touch football (n=45) | 15 (33.3%) | 29 (64.4%) | 5 (11.1%) |
| Gymnastics (n=39) | 10 (25.6%) | 11 (28.2%) | 21 (53.8%) |
| Athletics (n=36) | 17 (47.2%) | 10 (27.8%) | 11 (30.6%) |
| Basketball (n=30) | 10 (33.3%) | 17 (56.7%) | 4 (13.3%) |
| Tennis (n=30) | 11 (36.7%) | 13 (43.3%) | 8 (26.7%) |
| Oztag (n=28) | 5 (17.9%) | 20 (71.4%) | 5 (17.9%) |
| Australian Rules / AFL (n=24) | 5 (20.8%) | 16 (66.7%) | 6 (25.0%) |
| Cricket (n=21) | 4 (19.0%) | 12 (57.1%) | 8 (38.1%) |
| Yoga/pilates (n=16) | 5 (31.3%) | 10 (62.5%) | 1 (6.3%) |
| Volleyball (n=7) | 2 (28.6%) | 3 (42.9%) | 2 (28.6%) |
| Rugby (n=5) | 1 (20.0%) | 3 (60.0%) | 1 (20.0%) |
| Other (n=42) | 13 (31.0%) | 24 (57.1%) | 7 (16.7%) |

*Multiple responses could be selected
